# Differential transcript profiling through cDNA-AFLP showed complexity of rutin biosynthesis and accumulation in seeds of a nutraceutical food crop (*Fagopyrum* spp.)

**DOI:** 10.1186/1471-2164-13-231

**Published:** 2012-06-12

**Authors:** Nidhi Gupta, Pradeep Kumar Naik, Rajinder Singh Chauhan

**Affiliations:** 1 Department of Biotechnology & Bioinformatics, Jaypee University of Information Technology, Waknaghat, Solan, 173234 H.P, India

**Keywords:** Rutin, cDNA-AFLP, Transcript derived fragments, qRT-PCR

## Abstract

**Background:**

Buckwheat, consisting of two cultivated species *Fagopyrum tataricum* and *F. esculentum,* is the richest source of flavonoid rutin. Vegetative tissues of both the *Fagopyrum* species contain almost similar amount of rutin; however, rutin content in seed of *F. tataricum* are ~50 folds of that in seed of *F. esculentum*. In order to understand the molecular basis of high rutin content in *F. tataricum,* differential transcript profiling through cDNA-AFLP has been utilized to decipher what genetic factors in addition to flavonoid structural genes contribute to high rutin content of *F. tataricum* compared to *F. esculentum.*

**Results:**

Differential transcript profiling through cDNA-AFLP in seed maturing stages (inflorescence to seed maturation) with 32 primer combinations generated total of 509 transcript fragments (TDFs). 167 TDFs were then eluted, cloned and sequenced from *F. tataricum* and *F. esculentum*. Categorization of TDFs on the basis of their presence/absence (qualitative variation) or differences in the amount of expression (quantitative variation) between both the *Fagopyrum* species showed that majority of variants are quantitative (64%). The TDFs represented genes controlling different biological processes such as basic and secondary metabolism (33%), regulation (18%), signal transduction (14%), transportation (13%), cellular organization (10%), and photosynthesis & energy (4%). Most of the TDFs except belonging to cellular metabolism showed relatively higher transcript abundance in *F. tataricum* over *F. esculentum*. Quantitative RT-PCR analysis of nine TDFs representing genes involved in regulation, metabolism, signaling and transport of secondary metabolites showed that all the tested nine TDFs (Ubiquitin protein ligase, ABC transporter, sugar transporter) except MYB 118 showed significantly higher expression in early seed formation stage (S7) of *F. tataricum* compared to *F. esculentum*. qRT-PCR results were found to be consistent with the cDNA-AFLP results.

**Conclusions:**

The present study concludes that in addition to structural genes, other classes of genes such as regulators, modifiers and transporters are also important in biosynthesis and accumulation of flavonoid content in plants. cDNA-AFLP technology was successfully utilized to capture genes that are contributing to differences in rutin content in seed maturing stages of *Fagopyrum* species. Increased transcript abundance of TDFs during transition from flowers to seed maturation suggests their involvement not only in the higher rutin content of *F. tataricum* over *F. esculentum* but also in nutritional superiority of the former.

## Background

Buckwheat; *Fagopyrum* spp. is a pseudo-cereal multipurpose food crop used for both grains and greens with several medicinal and nutritional properties [1-3]. Genus *Fagopyrum* belongs to *Polygonaceae* and has 20 known species which mainly occur in the highlands of Euro-Asia [4-6]. Out of these, two cultivated species, *Fagopyrum esculentum* (common buckwheat) and *F. tataricum* (tartary buckwheat) are of high economic importance due to multiple uses such as a substitute for cereals in human consumption, as a vegetable crop, honey crop, and of ethno-botanical importance [7]. Significantly higher contents of flavonoids such as rutin and other polyphenols also add significance to the dietary value of buckwheat [8].

In tartary buckwheat, fagopyritols; mono-, di- and trigalactosyl derivatives of D-chiro-inositol account for 40% of total soluble carbohydrates compared to 21% in common buckwheat thus, helps in the treatment of diabetes [9]. Total flavonoids are relatively higher in tartary buckwheat (40 mg/g) compared to common buckwheat (10 mg/g) of which rutin is the major component [7]. Rutin a major flavonoid of medicinal value is found in higher quantities in buckwheat thus, considered as a major dietary source of rutin [1,2,8]. Tartary buckwheat seeds contain more rutin (about 0.8 to 1.7% DW) compared to common buckwheat seeds (0.01% DW) [8]. Due to the presence of proteins with high biological value (90%) and flavonoids with higher concentration in tartary buckwheat compared to common buckwheat, the former is considered an excellent food material with a potential for preventive nutrition [10]. But tartary buckwheat has a tightly adhering hull that makes it difficult to dehull and contains a bitter component that affects its palatability [1]. However, Rice-tartary is a type of tartary buckwheat (*F. tataricum*) with a non-adhering hull property, and can be a potential nutraceutical food source [11].

**Figure 1:**
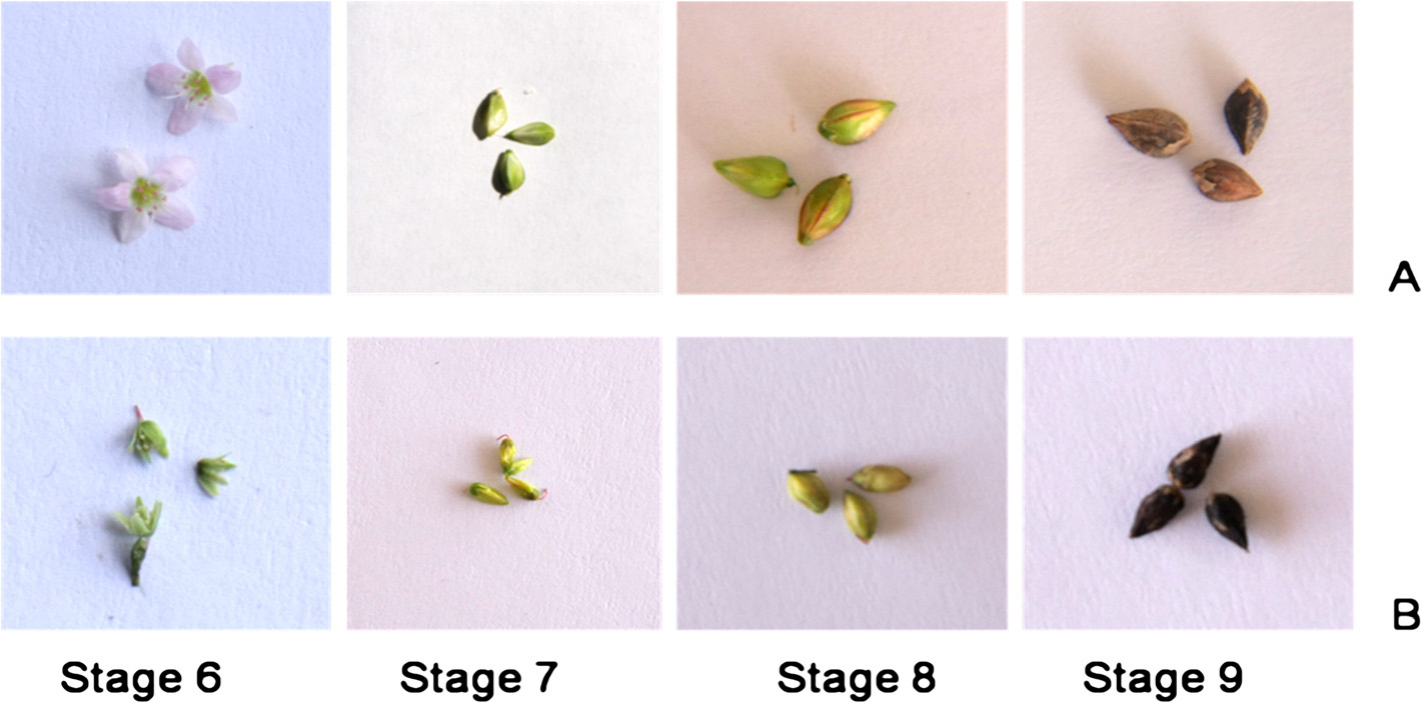
**Flowers to seed maturation stages of* Fagopyrum *spp., (A)* F. esculentum *(B)* F. tataricum*; Stage 6: Inflorescence; Stage 7: 12 DAP; Stage 8: 22 DAF; Stage 9: Mature seeds.**

**Figure 2:**
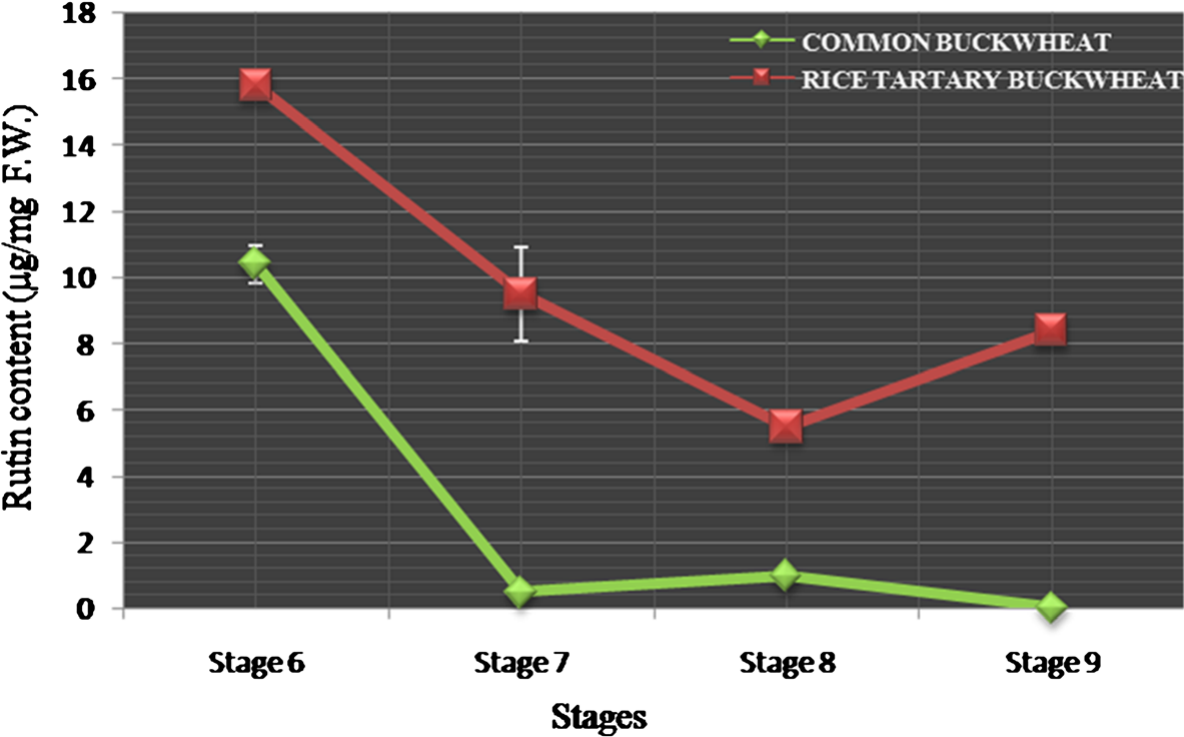
**Rutin content variation: rutin content variation during seed developmental stages of* Fagopyrum spp, *Rice-tartary Buckwheat (IC-329457), Common Buckwheat (IC-5408858).**

Molecular basis of nutritional superiority, particularly higher rutin content in *F. tataricum* compared to *F. esculentum*, is not fully understood. De-novo sequencing was used to understand molecular basis of morphological variations in the flowers of *Fagopyrum* species [12]. In addition, it has been observed that the flavonoid biosynthesis genes in *F. esculentum* were highly expressed in lower parts of plants than upper parts suggesting that flavonoids may be transported within a plant [13]. Anthocyanin content of *F. tataricum* has been correlated with the differential expression of flavonoid biosynthesis genes [14]. Comparative analysis of rutin content in different seed maturation stages of rice-tartary and tartary buckwheat compared to common buckwheat showed that all the post-flowering stages, S6, S7, S8 and S9 of rice-tartary contained 1.5, 31, 8, and 43x higher rutin content compared to common buckwheat, respectively [15]. Stages S6, S7, and S8 of rice-tartary contained higher rutin content even compared to tartary buckwheat; Figures 1 &2*.* Relatively higher expression of flavonoid pathway genes, phenylalanine ammonia lyase; PAL 4.3.1.24, chalcone synthase; CHS 2.3.1.74, chalcone isomerase; CHI 5.5.1.6 and flavonol synthase; FLS 1.14.11.23 were suggested to be responsible for higher rutin content in rice-tartary compared to common buckwheat [15]. However, increase in the expression of PAL, CHS, CHI and FLS genes did not occur concomitant to an increase in rutin content. Therefore, identification of additional genes, if any, was carried out to investigate molecular basis of high rutin content in flowering and post-flowering stages of *F. tataricum* compared to *F. esculentum.*

Flavonoid content in a particular tissue and developmental stage is largely influenced by different classes of regulatory genes, transporters, modifiers *etc*. in addition to structural genes of the flavonoid biosynthetic pathway [16-18]. Lack of whole genome sequence and non-availability of ESTs from developing seeds of *Fagopyrum* spp. prompted us to utilize cDNA-AFLP to decipher what genetic factors contribute to nutritional superiority of rice-tartary buckwheat compared to common buckwheat. Present study reports several differentially expressed transcript fragments representing genes involved in basic and secondary metabolism, transcription factors, transporters, etc., which were validated through qRT-PCR to associate their contribution in nutritional superiority of rice-tartary over common buckwheat.

## Results

### Identification and analysis of differentially expressed transcripts (TDFs)

cDNA-AFLP analysis on RNA samples from flower to mature seed stages of rice-tartary and common buckwheat with 32 primer pair combinations resulted in the identification of 42 clear and unambiguous fragments (TDFs). The TDFs ranged in sizes from 150–750 bp representing a total of 2,584 TDFs.

**Figure 3:**
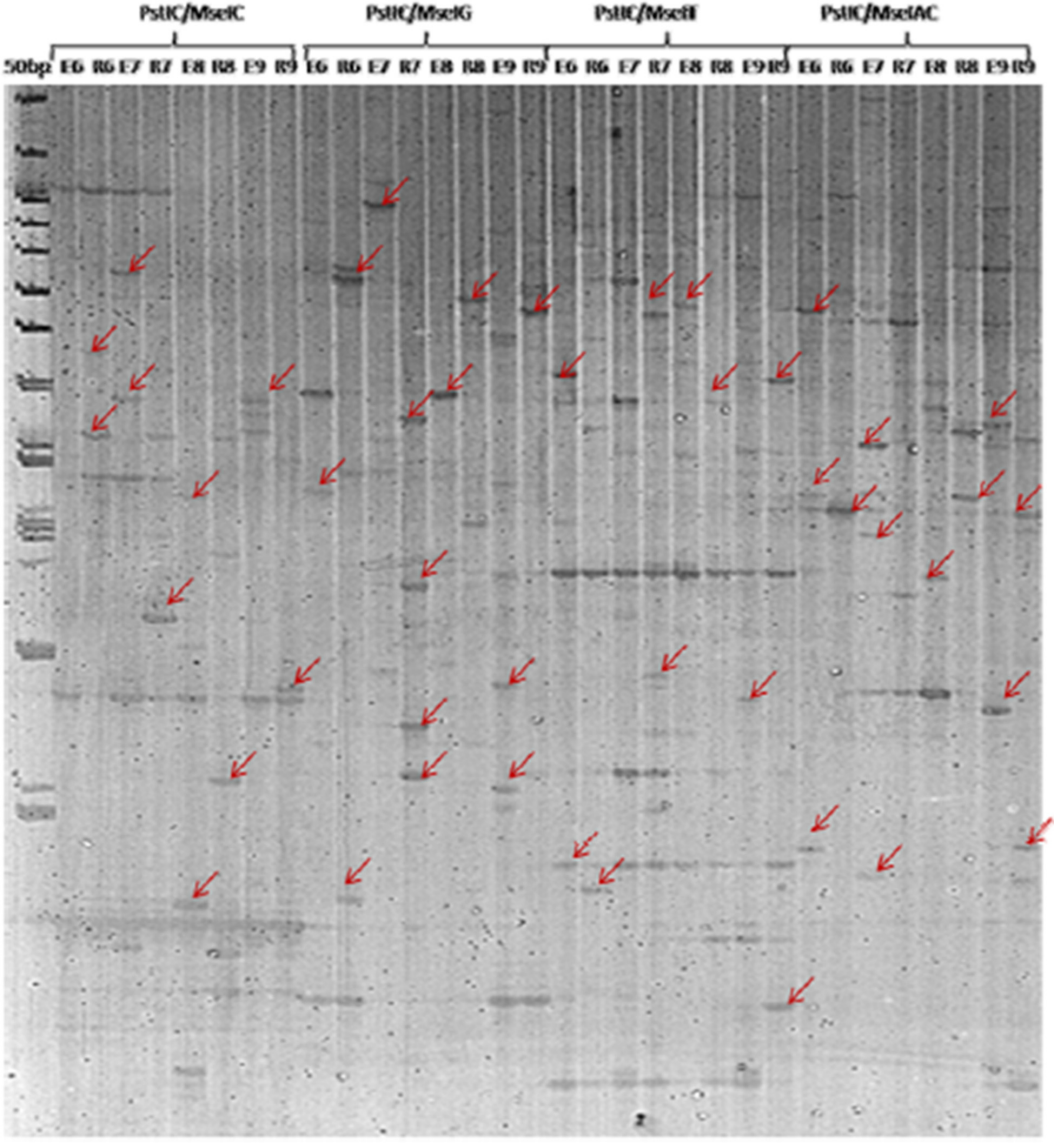
cDNA-AFLP patterns: Rice-tartary (R) vs Common Buckwheat (E) in seed maturation stages (6, 7, 8, and 9).

**Figure 4:**
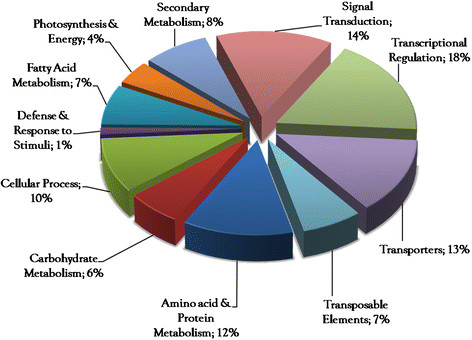
Functional classification of transcript derived fragments: Transcript derived fragments categorized into different categories.

A total of 167 differential TDFs based on presence/absence or differences in intensity were eluted from the gels, re-amplified and sequenced; Figure 3. DNA sequence of each TDF was assigned a putative biological function by checking against the Gene Bank database (BLASTN/BLASTX) as well as the TAIR database; Figure 4. TDFs represented genes controlling different biological processes such as general and secondary metabolism (33%), regulation (18%), signal transduction (14%), transportation (13%), cellular organization (10%), transposable elements (7%), photosynthesis (4%) and defense & response to stimuli (1%); Additional file 1: Table S1, Figure 4.

TDFs representing genes encoding alanine glyoxlate, methionine sulfoxide, fatty acid desaturase, KAS III, sucrose 6 phosphatase, ubiquitin protein ligases etc. were implicated in the biosynthesis of proteins & amino acids (12%), fatty acids (7%) and carbohydrates (6%). Similarly, 8% of TDFs corresponded to genes for key enzymes involved in secondary metabolism, including the flavonoid and anthocyanin biosynthesis. TDFs (10%) involved in cellular function included genes coding for pectin acyltransferases, proline rich extensins, glycine rich proteins, and arabinogalactan etc. TDFs corresponding to transporters (13%) included ABC transporters, auxin hydrogen symporter, sugar transporters, zinc and potassium transporters. Genes involved in signal transduction (14%) and regulation (18%), including Zn finger binding proteins, Leucine rich repeats calmodulin binding protein, protein kinases and transcription factors belongs to MYB and WRKY classes were also detected.

**Figure 5:**
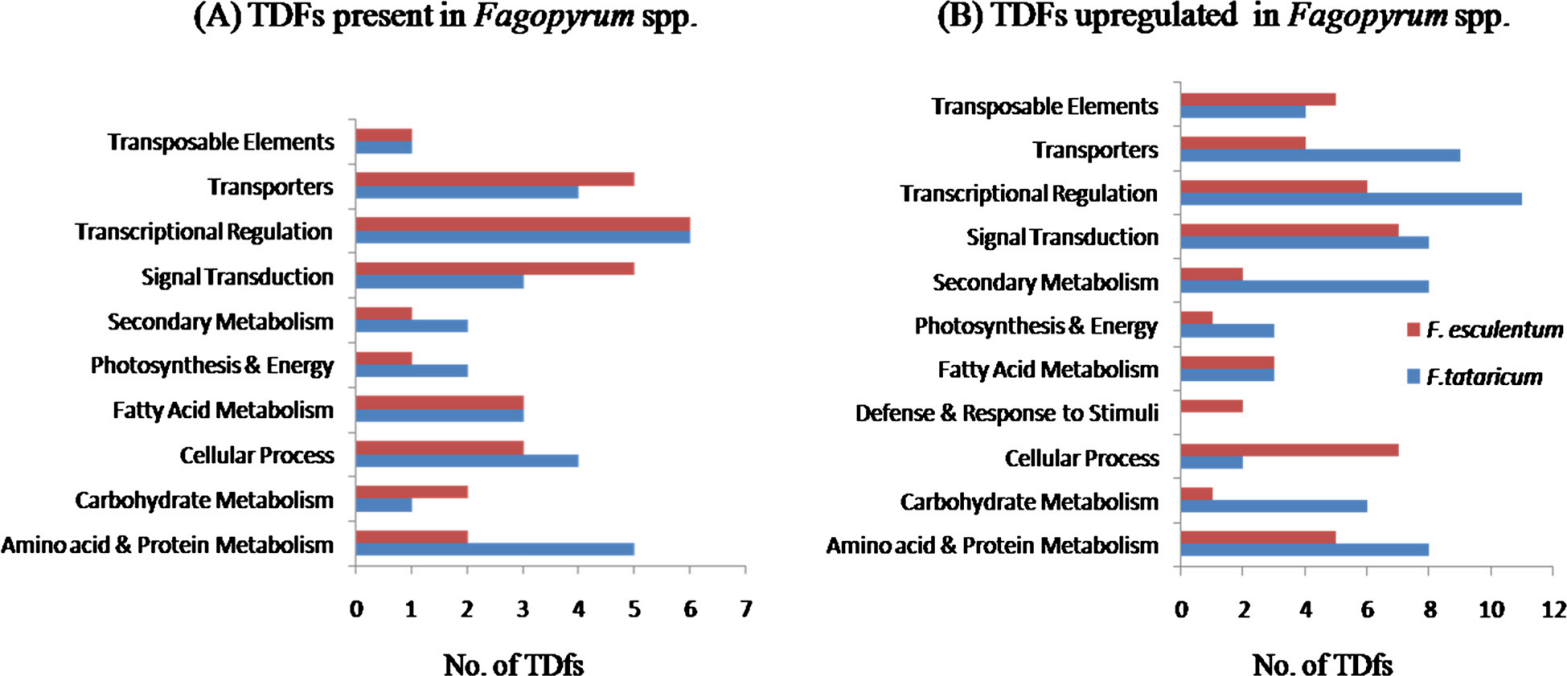
**Functional classification of differentially expressed TDFs in* Fagopyrum *species: On the basis of presence/absence and difference in intensities of TDFs.**

TDFs representing differentially expressed genes were classified into different categories on the basis of their presence/absence (qualitative variation) or differences in amount of expression (quantitative variation) between both the *Fagopyrum* species so as to infer whether TDFs belonging to a particular biological functional class are preferentially expressed in a particular *Fagopyrum* species; Figure 5A & B. TDFs representing genes involved in carbohydrate metabolism and signal transduction were relatively higher in number in *F. esculentum,* whereas TDFs representing genes involved in secondary metabolism, amino acid & protein metabolism, energy and photosynthesis were more in rice-tartary buckwheat; Figure 5A*.* Most of the TDFs belonged to genes for transporters, transcriptional regulation, secondary metabolism, photosynthesis & energy, carbohydrate, protein and amino acid metabolism and showed relatively increased transcript abundance in rice-tartary compared to common buckwheat; Figure 5B*.* Whereas, only TDFs for cellular metabolism showed relatively more transcript abundance in common buckwheat compared to rice-tartary buckwheat *.*

TDFs representing genes involved in transport of metabolites (ABC and sugar transporters, auxin hydrogen symporter), regulation of biosynthesis (MYB TF, Zn finger protein), metabolism of metabolites (ubiquitin protein ligases, extensin protein), signal transduction (calmodulin binding protein) and energy transfer (ATP CF0 subunit) were chosen for quantitative RT-PCR analysis in different seed maturing stages of both the *Fagopyrum* species ***.***

### Transcript quantification of selected TDFs in flower to seed maturation stages of *Fagopyrum* spp

**Figure 6:**
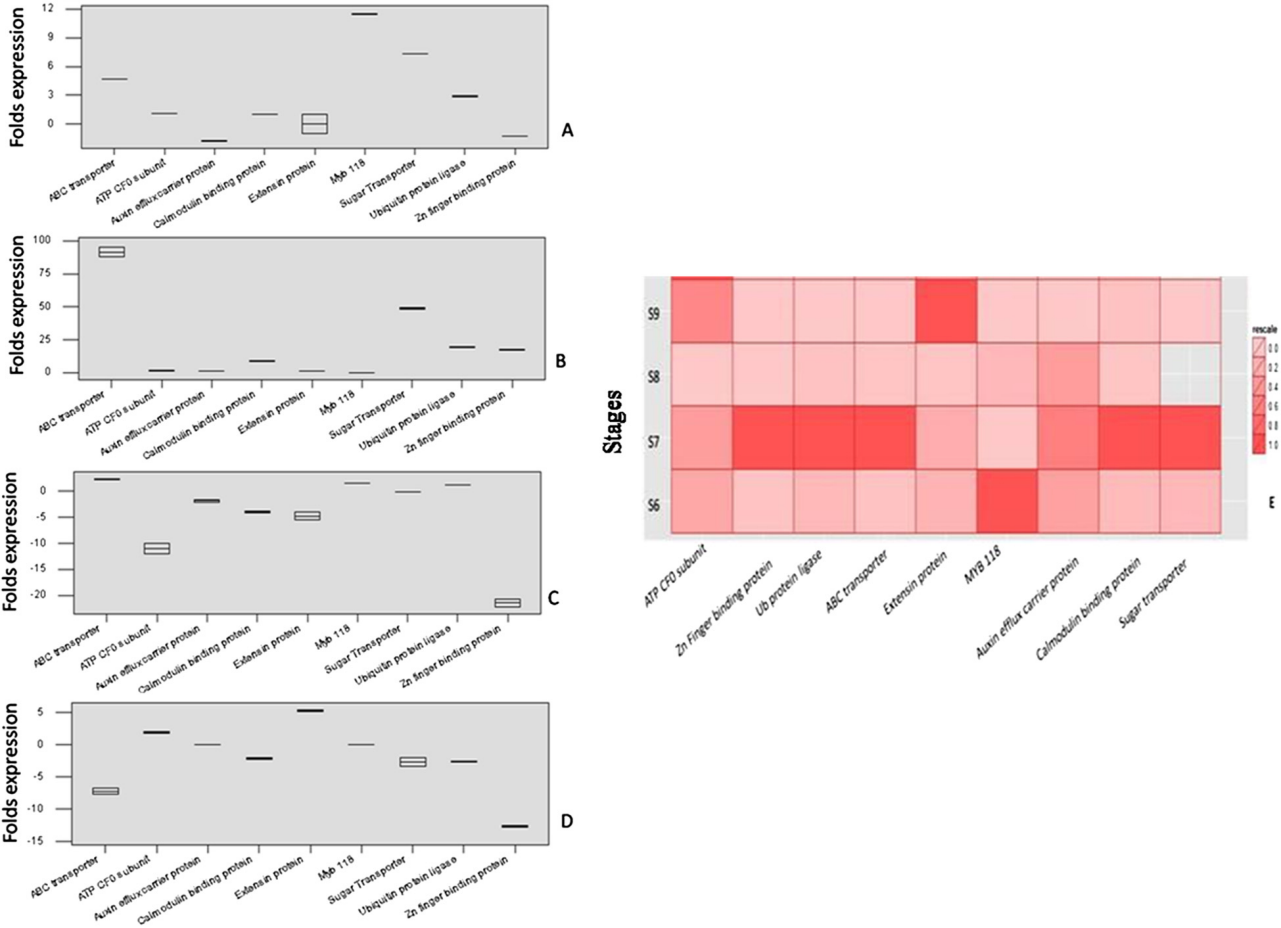
**Expression of TDFs in different tissues of* Fagopyrum *species.** Fold expression in rice-tartary buckwheat was calculated in comparison to their expression in different tissues of common buckwheat. A: Flowers (S6); B: Immature seeds (S7); C: Immature seeds (S8); D: Mature seeds (S9); E: Heat map of fold expression of TDFs expressed in seed maturation stages (S6-S9) of rice-tartary in comparison to common buckwheat.

Four TDFs representing genes for Ubiquitin protein ligase (JN982742), ABC transporter (JN982732), sugar transporter (JN982735) and MYB118 (JN982734) showed significantly increased transcript abundance in flowers (S6) of rice-tartary compared to common buckwheat with 2.86, 4.71, 7.36 and 11.42 folds expression, respectively; Figure 6A. Relatively higher abundance was observed for TDFs from genes for ABC transporter, sugar transporter, Ub protein ligase and Zn finger binding protein (JN982723) in immature seed stage (S7) of rice-tartary buckwheat with 95.38, 49.25, 18.92, 17.29 folds, respectively over common buckwheat; Figure 6B. On the other hand, 5 TDFs from genes encoding Zn finger binding protein, ATP CF0 subunit (JN982718), calmodulin binding protein, extensin (JQ003863) and auxin efflux (JN982731) showed relatively increased transcript abundance in the immature seeds (S8) of common buckwheat compared to rice-tartary buckwheat with 22.22, 11.94, 4.04, 3.89, and 1.64 folds higher abundance respectively. Two TDFs for ABC transporter (2.38 folds) and MYB118 (1.61 folds) showed relatively increased abundance in S8 of rice-tartary in comparison to common buckwheat; Figure 6C. In mature seeds (S9), the expression of TDFs Zn finger binding protein (12.54 folds), ABC transporter (6.78 folds), Ub protein ligase (2.54 folds), calmodulin binding protein (2.32 folds), and sugar transporter (2.07 folds) was found to be higher in common compared to rice-tartary buckwheat. Whereas, extensin and ATP CF0 subunit showed relatively higher expression in rice-tartary over common buckwheat in mature seeds (S9) with 5.42 and 2.09 folds, respectively; Figure 6D. No transcript was detected for MYB 118 and auxin efflux carrier protein in mature seeds of rice-tartary buckwheat*.* Therefore, the transcripts of ubiquitin protein ligases, ABC transporter and sugar transporter showed relatively higher expression in three stages of seed development, including flowers and immature seeds of rice-tartary compared to common buckwheat.

## Discussion

Understanding types and number of genes differentially expressed during seed maturation would help in discerning molecular mechanisms contributing to nutritional superiority, including high rutin content in the seeds of *F. tataricum* (rice-tartary buckwheat) over *F. esculentum*[16]. De-novo sequencing of transcripts from flowers of *Fagopyrum* species ( *F. tataricum* and *F. esculentum*) had shown that genes contributing to different biological processes are contributing to variations in the morphology of flowers in *Fagopyrum* species [12]. Accumulation of higher amounts of rutin during post-flowering stages of *F. tataricum* (rice-tartary buckwheat) over *F. esculentum* has also been observed [15]. Overall nutritional superiority of *F. tataricum* over other *Fagopyrum* spp has been found in the mature seeds as well as during seed maturation stages. For example, increase in fagopyritols content was shown from immature to mature seeds of buckwheat [19]. In addition, increase in the amount of sucrose and rutin was also reported during seed maturation of *Fagopyrum* species [15,20]. Molecular dissection of the genetic machinery contributing to nutritional differences in the seeds of *F. tataricum*, in particular the rice-tartary type over *F. esculentum* (common buckwheat) was undertaken through cDNA-AFLP since it is an open architecture technology for global transcriptional analysis in a non-model plant species [21,22].

Large numbers of TDFs with differential expression pattern were observed in different seed developmental stages of both the *Fagopyrum* species. TDFs with differential expression patterns belonged to genes involved in primary and secondary metabolism, transportation, signal transduction, gene regulation, photosynthesis & energy, defense and cellular processes in seed developmental stages of the *Fagopyrum* spp.; Additional file 1: Table S1. Out of 167 differential TDFs, only 22 showed 50–70% identity with the available floral transcriptome of both the *Fagopyrum* species [12], thereby, suggesting that most of the TDFs identified in the current study represented new genes. TDFs involved in transport, transcription, secondary metabolism, amino acid & protein metabolism, carbohydrate metabolism and photosynthesis were relatively higher in number and expression pattern in rice-tartary over common buckwheat*.* Higher expression of TDFs involved in secondary metabolism and transportation such as chalcone synthase, dihydroflavonol reductase, UDP glucosyl transferases, ABC transporters, MATE efflux carrier proteins, which are known to be involved in biosynthesis, accumulation and transportation of flavonoids, indicate their involvement in significantly higher flavonoid content in rice-tartary buckwheat [14,16,23]. Increased transcript abundance of TDF encoding Lys/His transporter in rice-tartary buckwheat was implicated for higher amount of histidine in this species [24]. TDFs involved in amino acid & protein metabolism (like Ub protein ligases, alanine glyoxylate amino transferases, cystein proteases), transcriptional regulation (MYB 118, MYB 112, GAMYB, histone acetyl transferases) and signal transduction (calmodulin binding protein, protein kinases, PEP carboxylase) were also found to be abundant in rice-tartary buckwheat. Most of these TDFs represent genes with their direct or indirect role in controlling the growth and development of seeds and/or their nutritional composition. On the basis of differential expression pattern of transcripts in rice-tartary and common buckwheat, the TDFs representing genes which have been implicated in biosynthesis, modification, regulation and transport of secondary metabolites [16,18,23] were chosen to investigate their role, through qRT-PCR analysis, in the biosynthesis of higher rutin content in the seeds of rice-tartary over common buckwheat.

The flavonoid content increase in buckwheat seedlings has been attributed to the increase in the concentration of sucrose [25]. In addition, sugars also act as developmental signals regulating seed maturation and accumulation of flavonoids in plants such as *Arabidopsis, V. vinifera*[26-28]. Exponential increase in the transcript of a sugar transporter (JN982735) from flowers (S6) to immature seeds (S7) of rice-tartary compared to common buckwheat suggests its contribution in higher content of flavonoids and fagopyritols in the seeds of rice-tartary buckwheat. Relatively increased transcript abundance of auxin efflux carrier protein (JN982731) in different seed maturation stages (S8 & S9) of *F. esculentum* suggests its negative role in the biosynthesis of flavonoids, which are present in lower amounts in different growth stages of *F. esculentum*[15]. Flavonoids have been implicated as inhibitors of auxin transport in *Arabidopsis*[18,29]. The ABC and MATE classes of transporters are known to be involved in the transport of flavonoids from cytosol into vacuoles [16,23,30]. In present study the abundance of TDF ABC transporter (JN982732) was relatively higher in flowering to seed maturation stages, S6 (4.7x), S7 (95.4x), and S8 (2.4x) of rice-tartary buckwheat compared to common buckwheat suggested that this gene might be playing a key role in the transport of flavonoids (rutin, quercetin and quercitrin) in rice-tartary buckwheat. Also, it has been shown that biosynthesis of flavonoids takes place in lower parts of *Fagopyrum* spp. and then gets transported to upper parts [13].

Significantly higher expression of 4 TDFs representing genes for ubiquitin protein ligase, ABC transporter, sugar transporter and calmodulin binding protein in S7 of rice-tartary buckwheat compared to *F. esculentum* suggests their major involvement in nutritional superiority of rice-tartary buckwheat. The calmodulin binding proteins regulate diverse cellular processes by interacting with other proteins and help in secondary metabolism by acting as secondary messengers through signal transduction [31,32]. In addition, calmodulin proteins are also known to induce anthocyanin biosynthesis in *V. vinifera*[33]. Extensins, the major structural proteins in plant cell wall play important role in various biological processes such as embryo development, root hair growth, seed coat development, defense, *etc*. [34,35]. Increased transcript abundance of extensin protein (JQ003863) in mature seeds of rice-tartary buckwheat in comparison to common buckwheat may contribute to development of seeds of both the *Fagopyrum* species.

Transcription factors are known to play important role in various seed development processes and regulation of secondary metabolism [36-38]. Zn finger binding proteins (JN982723) have been implicated in regulation of important biological processes such as flower and seed development, seed germination, stress tolerance in *Arabidopsis*[39,40]. Expression of a TDF representing a gene for Zn finger binding protein was relatively higher in seed developing stages S8 and S9 of common buckwheat compared to rice-tartary buckwheat. Transcript of a TDF encoding for another transcription factor MYB 118 (JN982734) was high in the flowers (S6) of rice-tartary compared to common buckwheat.

Ub protein ligases (JN982742) are known to regulate various biological processes like photomorphogenesis, hormonal response, senescence, floral, embryo and seedling development through degradation of proteins as well as regulates phenylpropanoid pathway during UV stress and sugar signaling during seedling development [41-43]. Relatively higher expression of Ub protein ligase in immature seeds (S7) of rice-tartary buckwheat has been observed in the present study. Significantly higher expression of most of the selected TDFs during early seed formation stage (S7) of rice-tartary buckwheat (Figure 6E) reflects their biological importance in maintaining higher amounts of rutin, which otherwise drops significantly in the same stage of *F. esculentum*.

## Conclusions

The present study concludes that in addition to structural genes, the other classes of genes such as regulators, modifiers and transporters are equally important in contributing to higher flavonoids content and nutritional superiority of *F. tataricum* (rice-tartary buckwheat) over *F. esculentum*. Increased transcript abundance of selected TDFs in rice-tartary buckwheat during early seed maturation stage (S7) i.e. the transition from flowers to seed formation also reflects their contribution not only in higher rutin content but also in other biological processes which are contributing to overall nutritional differences between both the *Fagopyrum* species. In summary, the cDNA-AFLP technology was successfully utilized to identify genes that are differentially expressed in seed maturation stages of two *Fagopyrum* spp. Therefore, identification of several genes representing regulators, modifiers or transporters, has opened up avenues to investigate their precise role in contributing to higher rutin content as well as overall nutritional superiority of rice-tartary over common buckwheat.

## Methods

### Plant material

Seeds of *F. tataricum* (rice-tartary buckwheat) and *F. esculentum* (common buckwheat) were procured from the National Bureau of Plant Genetic Resources (NBPGR), Regional Research Station, Phagli, Shimla (H.P. India), India and germinated in a potting mixture consisting of soil and vermiculite in a ratio of 1:1. Seedlings were grown under controlled conditions of light (intensity 300–1400 lx), temperature (25 ± 2°C), humidity (≈ 70%), and photoperiod of 14 h day/10 h night. Samples of different seed developmental stages *i.e.* from flowering to seed maturation (Figure 1) were collected (June to September). Samples were harvested between 9 and 10 AM, immediately frozen in liquid nitrogen and stored at −80°C for further use in isolation of genomic DNA and mRNA.

### Isolation of genomic DNA, RNA and cDNA synthesis

Genomic DNA was isolated from leaf samples of both the *Fagopyrum* spp. by following the protocol of Murray and Thompsan [44]. Total RNA was isolated from flowers, immature seeds and mature seeds of both the *Fagopyrum* spp. by using Raflex RNA isolation kit (GeNei^TM^, Bangalore, India) by following manufacturer's instructions. Quality of DNA and RNA was checked by 1% (w/v) ethidium bromide-stained agarose gel and from the absorbance spectrum at wavelengths 260 nm and 280 nm.

First strand cDNA was synthesized from 5 μg of RNA-free from DNA (RNA preparation was treated with 2U of DNAse I), reverse transcribed by using M-MuLV reverse transcriptase (GeNei^TM^) and an oligo-dT _(12–18)_ primer. Double-stranded cDNA was synthesized according to a standard double-stranded cDNA synthesis protocol [45], using DNA polymerase I (*Escherichia coli*) and T4 DNA ligase (New England Biolabs Inc., Beverly, MA).

### cDNA-AFLP analysis

About 500 ng of double stranded cDNA was subjected to standard AFLP template production according to Vos et al. [46]. cDNA was digested with restriction enzymes *Mse*I and *Pst*I(NEB, England). Digested products were then ligated to adapters with sequences as follows: *Mse*I adapter, 5’-GACGATGAGTCCTGAG-3’, 3’-TACTCAGGACTCAT-5’; *Pst*I adaptors 5’-CTCGTAGGACTGCGTACATGCA-3’, 3’-TGTACGCAGTCTAC-5’. Adapter-ligated DNA served as a template for pre-amplification, with PCR parameters of 30 cycles of 30 s at 94°C, 60 s at 56°C, and 60 s at 72°C. The diluted (30-fold) amplified products were used as the template for selective amplification. Equal amounts of pre-amplified products were amplified with primers having selective nucleotides at the 3’ end in a total volume of 20 μl; Additional file 2: Table S2. First selective amplification cycle consisted of 30 s at 94°C, 30 s at 65°C, and 60 s at 72°C; annealing temperature was lowered by 0.7°C per cycle during the next 12 cycles, followed by 23 cycles at 94°C for 30s, 56°C for 30 s, and 72°C for 60s. All PCR reactions were carried out in Applied Biosystem model no-9902 Veriti thermal cycler. To each PCR product 7.5 μl of formamide dye (98% formamide, 10 mM EDTA, 0.005% xylene cyanol FF, and 0.005% bromophenol blue) was added, and 7 μl of each sample was loaded onto a pre-warmed 6% polyacrylamide gel using 1X Tris–borate–EDTA (TBE) buffer. Electrophoresis was then run for 2.5 h at 65 W and the gels were silver stained using a silver staining kit (Promega cat. #Q4132, Madison, WI), following the manufacturer's instructions.

### Transcript-derived fragment (TDF) isolation and re-amplification

Differentially expressed TDFs based on presence, absence or differences in intensity were cut with a sharp blade from the gel with care to avoid any contaminating fragment(s), eluted in 50 μl of sterile double distilled water, incubated at 95°C for 15 min and then hydrated overnight at 4°C. An aliquot of 2 μl was used for re-amplification in a total volume of 25 μl, using the same set of corresponding selective primers and PCR conditions as used for the selective amplification, except that an annealing temperature of 56°C for 35 cycles was used. PCR products were resolved in a 2% agarose gel; each single band was isolated and eluted using the Genaxy DNA gel extraction kit (Genaxy Biosciences Inc., USA). The reproducibility of cDNA AFLP was verified by repeating the experiment twice.

### Cloning and sequencing of TDFs

Eluted TDFs were cloned into the plasmid pGEM-Teasy® vector (Promega Corp., Madison, WI) following the manufacturer's protocol and then sequenced. Sequences of TDF (with vector sequences trimmed off, where plasmid was used as the template) were then analyzed for their homology against the publicly available non-redundant genes/ESTs/Transcripts in the database (http://www.ncbi.nlm.nih.gov/BLASThttp://www.arabidopsis.org/Blast) using BLASTN and BLASTX algorithms. TDFs were also checked for putative function against *Arabidopsis* database using FASTA tool (http://www.arabidopsis.org/cgi-bin/fasta/nph-TAIRfasta.pl) developed by TAIR, [47].

### Real-time RT-PCR analysis

Specific primer pairs were designed for TDFs; Additional file 3: Table S3 and tested by real time RT-PCR. Primers specific for buckwheat 26 S *rRNA* and Histone H3 were used for the normalization of reactions. Real-time PCR reactions were performed in duplicate on a CFX 96 system (Bio-Rad Laboratories; Hercules,CA) with the iScript one step RT PCR Kit (Bio-rad). PCR protocol was as follows: denaturation for 5 min at 94°C, followed by 40 cycles each of denaturation for 20 s at 94°C, annealing for 30 s at 55–61°C, and one elongation step for 20 s at 72°C. Significance of differences between treatments was statistically analyzed. Whisker plots were drawn for qRT-PCR data to determine folds expression of TDFs in *F. tataricum* compared to *F. esculentum* by using MINITAB-14.

## Competing interests

The authors declare no competing interests.

## Authors' contributions

NG carried out the experiments, analyzed the data and drafted the manuscript. PKN participated in data analysis. RSC proposed and supervised the research, helped to draft and reviewing the manuscript. All authors read and approved the final manuscript.

## Supplementary Material

Additional file 1 **Table S1.** Sequence homology: Functional characterization of transcript derived fragments (TDFs) based on BLAST X and TAIR FASTA analysis. Click here for file

Additional file 2 **Table S2.** Primers List: Primers used for cDNA-AFLP analysis. Click here for file

Additional file 3 **Table S3.** Primers List: Primers used for real time qRT-PCR analysis. Click here for file
