# Supplementary material for: Differential transcript profiling through cDNA-AFLP showed complexity of rutin biosynthesis and accumulation in seeds of a nutraceutical food crop (Fagopyrum spp.)
Source: BMC Genomics. 2012 Jun 12;13:231. doi: 10.1186/1471-2164-13-231 (PMC3441755; doi:10.1186/1471-2164-13-231)
Supplement: Additional file 1 — Table S1. Sequence homology: Functional characterization of transcript derived fragments (TDFs) based on BLAST X and TAIR FASTA analysis. [file 1471-2164-13-231-S1.doc]

| **Sequence Name**  **Additional File 1 Sequence homology**: Functional characterization of transcript derived fragments (TDFs) based on BLAST X and TAIR FASTA analysis | **Accession Number** | **Homology to gene (Genebank ID)** | **Organism** | **E-value Score** | **Function** | **Metabolic Pathway** |
| --- | --- | --- | --- | --- | --- | --- |
| **Secondary Metabolism** | | | | | | |
| TDF 4 | JN982719 | Dihydroflavonol 4 reductase (ACG42172.1) | *Zea mays* | 2e-05 | Anthocyanin Biosynthesis | Anthocyanin biosynthesis |
| TDF 14 | JN982729 | Cinnamoyl CoA reductase (AAY86360.1) | *Acacia auriculiformis X Acacia mangium* | 1e-12 | Key enzyme of monolignoids | Lignin biosynthesis |
| TDF 19 | JN982733 | Chalcone synthase (ACH70135.1) | *Fagopyrum tataricum* | 4e-27 | Key enzyme of flavonoid biosynthesis | Flavonoid biosynthesis |
| TDF 32 | JN982746 | Cinnamoyl CoA reductase family (XP_002526624.1) | *Ricinus communis* | 0.014 | Key enzyme of monolignoids | Lignin biosynthesis |
| TDF 95 | JQ417192 | NAD(P)-Binding Rossamann Fold (AT1G09500) | *Arabidosis thaliana* | 0 | Cinnamoyl CoA dehydrogenase activity | Phenylpropanoid biosynthesis, lignin biosynthesis |
| TDF 13 | JN982728 | Cytochrome P450 family (AT5G52320) | *Arabidosis thaliana* | 1.8e-06 | Catalyze the oxidation of various intermediates | Limonene and pinene degradation, Stilbenoid, diarylheptanoid and gingerol biosynthesis, Biosynthesis of secondary metabolites |
| TDF 116 | JQ417191 | UDP Glucosyl Transferases (AT5G03490) | *Arabidosis thaliana* | 1.3e-189 | Transfer of glucose to flavonol | Flavone and flavonol biosynthesis (ath00944), Glycosylation regulates transportation, solubility |
| **Transporters** | | | | | | |
| TDF 6 | JN982721 | Zinc Transporter (XP_002517414.1) | *Ricinus communis* | 5e-04 | Transportation of Zn ions | Cation transmembrane transporter activity, transportation of Zn (micronutrient) from soil to roots and then to various plant parts |
| TDF 16 | JN982731 | Auxin efflux carrier protein / Auxin:Hydrogen symporter (NP_197014.1) | *Arabidopsis lyrata* | 0.007 | Transportation of auxins | Regulation of auxin homestasis within plant cells |
| TDF 18 | JN982732 | ATP binding cassette transporter putative (XP_002516304.1) | *Ricinus communis* | 9e-05 | Transportation of flavonoids | Transportation of flavonoids from cytosol to vacuoles |
| TDF 21 | JN982735 | Sugar transporter protein (NP_001191180.1) | *Zeamays* | 3.2 | Carbohydrate transmembrane transport, proton transport | Transportation of sugars to cells for growth and development, and for synthesis of storage compounds |
| TDF 30 | JN982744 | Putative high affinity potassium transporter (BAD31835) | *Oryza sativa* | 1e-06 | Transportation of K+ ions | K^+^ uptake from soils into roots and from roots to different cells within plants |
| TDF 35 | JQ003853 | MATE efflux family protein (AT1G61890) | *Arabidopsis thaliana* | 6.6e-06 | Transmembrane transport of metabolites | Vacuolar sequestration of flavonoids, efflux of toxic compounds etc |
| TDF 36 | JQ003854 | Transmembrane amino acid Lys/His transporter (AT1G47670.1) | *Arabidopsis thaliana* | 6.5e-05 | Transportation of amino acids Lys/His | Transportation of amino acids during seed germination and grain filling |
| TDF 37 | JQ003855 | Glucose-6-phosphate antiporter/ triose-phosphate transmembrane transporter (AT5G46110.3) | *Arabidopsis thaliana* | 1.4e-50 | Transportation of glucose-6-phosphate | Glucose 6-P transportation in plastids for starch biosynthesis and pentose phosphate pathway |
| TDF 38 | JQ003856 | Sec23/Sec24 protein transporter (AT4G32640) | *Arabidopsis thaliana* | 0 | ER to golgi vesicle mediated protein transport | Protein processing in endoplasmic reticulum (ath04141) |
| **Photosynthesis and energy** | | | | | | |
| TDF 24 | JN982738 | NADH dehydrogense subunit F (ABP88019.1) | *Scoparia dulcis* | 5.5 | Energy, ATP synthase copled electron transport | Oxidative phosphorylation |
| TDF 3 | JN982718 | Putative ATP CF0 subunit (CAO02552.1) | *Vigna unguiculata, Fagopyrum esculentum* | 3e-03 | Oxidative phosphorylation | Translocation of protons across the membrane |
| **Fatty Acid Biosynthesis** | | | | | | |
| TDF 39 | JQ003857 | GroES-like zinc-binding dehydrogenase (AT1G32780.1) | *Arabidopsis thaliana* | 1.2e-06 | Alcohal dehydrogenase activity | Fatty acid metabolism (ath00071) |
| TDF 123 | JQ417190 | Phosphatidic acid phosphatase (AT5G03080.1) | *Arabidopsis thaliana* | 1.5e-62 | Lipid synthesis by dephosphlylating phosphatidic acid | N-Glycan biosynthesis (ath00510) |
| TDF 11 | JN982726 | ACC Biotin containing subunit (AAC49114.1) | *Arabidopsis thaliana* | 1e-07 | Carboxylation of Acetyl CoA to Malonyl CoA | Fatty acid biosynthesis. |
| TDF 40 | JQ003858 | 3-ketoacyl-CoA synthase (AT3G52160.1) | *Arabidopsis thaliana* | 8.4e-05 | Condensation of a malonyl-ACP with an acyl acceptor | Fatty acid elongation (ath00062) |
| TDF 29 | JN982743 | Lipoxygenase (AAF15296.2) | *Phaseolus vulgaris* | 5e-05 | catalyzes the addition of molecular oxygen to polyunsaturated fatty acids to produce an unsaturated fatty acid hydroperoxide | In defense against pathogens, fruit ripening, nitrogen storage, nodule development etc. |
| TDF 26 | JN982740 | Phospholipase C6 (NP_190430.2) | *Arabiodopsis thaliana* | 1.2 | Cellular regulation, lipid metabolism, membrane remodeling | Hydrolyze phospholipids for the release of secondary messenger that participate in different signal transduction pathway |
| TDF 43 | JQ003861 | GDSL-like Lipase/Acylhydrolase (AT1G20130.2) | *Arabiodopsis thaliana* | 0 | Hydrolysis of ester bonds | Lipid metabolism |
| **Cellular process** | | | | | | |
| TDF 10 | JN982725 | Nodulin (NP_194482.1) | *A.thaliana* | 1e-05 | nodulin like protein L2 | Reproduction of plants, symbiosis |
| TDF 41 | JQ003859 | Actin binding (AT5G07740) | *Arabiodopsis thaliana* | 3e-201 | Rearrangement of the actin cytoskeleton | Regulates cell growth and morphology |
| TDF 44 | JQ003862 | Pectin acetylesterase family protein (AT3G62060.1) | *Arabiodopsis thaliana* | 1e-141 | Cell wall modification | Hydrolyze acetyl esters in pectin |
| TDF 45 | JQ003863 | Proline-rich extensin-like family protein (AT5G19810.1) | *Arabiodopsis thaliana* | 8.9e-05 | Major ptoteins in plant cell wall | Important role in various biological processes such as embryo development, root hair growth, seed coat development, defense etc. |
| TDF 33 | JN982747 | ribosomal protein L2 (ATCG01310.1) | *Arabidopsis thaliana* | 3.3e-06 | Structural constituent of ribosome | Ribosome (ath03010) |
| TDF 47 | JQ003865 | 75K gamma secalin (ADP95516.1) | *Triticum aestivum* | 0.045 | Nutrient reservoir activity | Storage protein |
| **Protein metabolism/ Amino acid biosynthesis** | | | | | | |
| TDF 48 | JQ003866 | Eukaryotic aspartyl protease (AT4G16563.1) | *Arabidopsis thaliana* | 0.049 | Proteolysis | Cleavage of specific dipeptide bonds |
| TDF 5 | JN982720 | Cysteine protease (AAP41486.1) | *Anthurium andraeanum* | 2e-05 | Proteolytic activity | Involved in protein maturation, degradation, and protein rebuilt in response to different external stimuli |
| TDF 28 | JN982742 | Ubiquitin protein ligase (EEE30515) | *Ricinus communis* | 2e-04 | Protein degradation | Protein degradation |
| TDF 9 | JN982724 | Pyridoxal-5'-phosphate-dependent Threonine synthase (AT1G72810.1) | *Arabidopsis thaliana* | 1e-84 | Threonine biosynthesis process | Glycine, serine and threonine metabolism Vitamin B6 metabolism (ath00750) |
| TDF 51 | JQ003869 | Alanine:glyoxylate aminotransferase (AT4G39660.1) | *Arabidopsis thaliana* | 1.9e-43 | Glyoxylate transaminase activity | Alanine, aspartate and glutamate metabolism (ath00250), Glycine, serine and threonine metabolism (ath00260) |
| TDF 52 | JQ003870 | Peptidylprolyl Isomerase (AAF75383.1) | *Arabidopsis thaliana* | 3e-04 | Isomerisation of dipeptide bonds | Protein scaffolding |
| **Transcriptional regulation** | | | | | | |
| TDF 8 | JN982723 | Zinc finger family protein (XP 0028927381.1) | *Arabidopsis lyrata* | 0.003 | Transcriptional regulation | Inactivation of transcription, flower and seed development, seed differentiation, stress tolerance |
| TDF 22 | JN982736 | Histone acetyl transferase (NP_001105145.1) | *Zeamays* | 7e-04 | Transcriptional regulation | Gene regulation, acylation of histone and non-histone proteins like transcription factors, nuclear receptors to facilitate gene expression |
| TDF 7 | JN982722 | GA MYB Like 2 (ABM53274.1) | *Solanum lycopersicum* | 4e-04 | Important role in pollens and anther development in response to gibberellic acid | Mediate GA signaling |
| TDF 20 | JN982734 | MYB 118 (XP_002877072.1) | *Arabidopsis lyrata* | 3e-04 | Transcriptional regulation | Embryogenesis and seed development & maturation in *Arabidopsis* |
| TDF 53 | JQ003871 | MYB112, AtMYB112 | myb domain protein (AT1G48000.1) | *Arabidopsis thaliana* | 6.3e-129 | Transcription factors | Transcriptional regulation |
| TDF 34 | JQ003852 | FH5; formin homolog protein 5 (AAS93430.1) | *Arabidopsis thaliana* | 4.1 | Transcriptional regulation | Mediate ubiqutination, signal transduction, regulation of cell cycle, transcriptional regulation |
| **Carbohydrate Metabolism** | | | | | | |
| TDF 31 | JN982745 | Starch branching enzyme 3 (ABU41261) | *Oryza sativa* | 1e-04 | Starch biosynthesis by introduction of branch points | Starch metabolism |
| TDF 54 | JQ417183 | UDP-D-apiose/UDP-D-xylose synthase (AT2G27860.1) | *Arabidopsis thaliana* | 7e-05 | Sugar biosynthesis | Amino sugar and nucleotide sugar metabolism (ath00520), hemicelluloses synthesis |
| TDF 63 | JQ417189 | Sucrose-6F-phosphatase (AT3G52340.1) | *Arabidopsis thaliana* | 5.9e-55 | Sucrose biosynthesis | Final step in sucrose biosynthesis |
| TDF 42 | JQ003860 | GDP Mannose 4,6 dehydrogense | *Arabiodopsis thaliana* | 1.8e-25 | metabolism | Cell wall carbohydrate synthesis, protein glycosylation |
| **Signal Transduction** | | | | | | |
| TDF 15 | JN982730 | Calmodulin dependent protein kinase (AAG01179.1) | *Zeamays* | 2.1 | Acting as a secondary messengers through signal transduction | Regulate diverse cellular processes by interacting with other proteins |
| TDF 50 | JQ003868 | GTP binding protein subunit (XP_002519124) | *Ricinus communis* | 2e-04 | Signal transduction | Control of plant growth, differentiation and development in responses to biotic and abiotic stress |
| TDF 23 | JN982737 | Protein kinases (ABA9926) | *Oryza sativa* | 6e-06 | Catalyze the reversible transfer of the y-phosphate from ATP to amino acid side chains of proteins. | Regulating cellular division, cell differentiation and morphogenesis |
| TDF 25 | JN982739 | Receptor like protein kinase (ACM89561.1) | *Glycine max* | 0.94 | Signal Transduction | Control protein activity and cellular signalling |
| TDF 46 | JQ003864 | Leucine-rich repeat (LRR) family protein (AT3G05990.1) | *Arabidopsis thaliana* | 3e-116 | Signal Transduction | Plant LRR proteins involved in such diverse processes as pollen tube growth, root development, Ran GTPase activation, transcription regulation and meristem cell organization |
| **Defense and Response to stimulus** | | | | | | |
| TDF 55 | JQ417184 | TIR disease resistance protein (NP_001185276) | *Arabidopsis thaliana* | 7e-05 | Defense response | Defense against pathogens |
