# Supplementary material for: Differential transcript profiling through cDNA-AFLP showed complexity of rutin biosynthesis and accumulation in seeds of a nutraceutical food crop (Fagopyrum spp.)
Source: BMC Genomics. 2012 Jun 12;13:231. doi: 10.1186/1471-2164-13-231 (PMC3441755; doi:10.1186/1471-2164-13-231)
Supplement: Additional file 2 — Table S2. Primers List: Primers used for cDNA-AFLP analysis. [file 1471-2164-13-231-S2.doc]

**Additional File 2 Primers List:** Primers used for cDNA-AFLP analysis

| **Pre-selective primers** | **5′-Sequence-3′** |
| --- | --- |
| Pst I (00) | GAC TGC GTA CAT GCA G |
| MseI (00) | GAC GAT GAG TCC TGA GTA A |
| **Selective primers** | **5′-Sequence-3′** |
| Pst I (T) | GAC TGC GTA CAT GCA GT |
| Pst I (C) | GAC TGC GTA CAT GCA GC |
| Pst I (A) | GAC TGC GTA CAT GCA GA |
| Pst I (G) | GAC TGC GTA CAT GCA GG |
| Pst I (CT) | GAC TGC GTA CAT GCA GCT |
| Pst I (GA) | GAC TGC GTA CAT GCA GGA |
| Pst I (GG) | GAC TGC GTA CAT GCA GGG |
| Pst I (CA) | GAC TGC GTA CAT GCA GCA |
| Pst I (AT) | GAC TGC GTA CAT GCA GAT |
| Pst I (CAA) | GAC TGC GTA CAT GCA GCA A |
| Pst I (CTA) | GAC TGC GTA CAT GCA GCT A |
| Msel (C) | GAC GAT GAG TCC TGA GTA AC |
| MscI (G) | GAC GAT GAG TCC TGA GTA AG |
| MseI (A) | GAC GAT GAG TCC TGA GTA AA |
| MseI (T) | GAC GAT GAG TCC TGA GTA AT |
| MseI (CA) | GAC GAT GAG TCC TGA GTA ACA |
| MseI (GA) | GAC GAT GAG TCC TGA GTA AGA |
| MseI (TT) | GAC GAT GAG TCC TGA GTA ATT |
| MseI (AC) | GAC GAT GAG TCC TGA GTA AAC |
| MseI (AT) | GAC GAT GAG TCC TGA GTA AAT |
| MseI (AG) | GAC GAT GAG TCC TGA GTA AAG |
| MseI (TA) | GAC GAT GAG TCC TGA GTA ATA |
| MseI (CAA) | GAC GAT GAG TCC TGA GTA ACA A |
