# Supplementary material for: Differential transcript profiling through cDNA-AFLP showed complexity of rutin biosynthesis and accumulation in seeds of a nutraceutical food crop (Fagopyrum spp.)
Source: BMC Genomics. 2012 Jun 12;13:231. doi: 10.1186/1471-2164-13-231 (PMC3441755; doi:10.1186/1471-2164-13-231)
Supplement: Additional file 3 — Table S3. Primers List: Primers used for real time qRT-PCR analysis. [file 1471-2164-13-231-S3.doc]

**Additional File 3 Primers List:** Primers used for real time qRT-PCR analysis

| Gene Name | Forward Primer Sequence (5’-3’) | Reverse Primer Sequence (5’3’) |
| --- | --- | --- |
| JQ003863 | GGGCCTCATTCTTCAACAGG | CCATAGCGAAGTAGTACCGTGAG |
| JN982731 | CACCCCTTTATAAAAGACTTCTTCC | AATCAAGCATCAAAGCTATCAACTC |
| JN982742 | ACAAACAGGTACCATAAAGCCCTAC | GGGCACTATACCTGAGTAACACCTA |
| JN982718 | CTACTGGACTCTCGCCATCTAGG | CTTAGTAGCCAGAGGAAAAGAAAGC |
| JN982730 | CCCACATTACATAACACTCACATCT | GAGACTAGGCCATACACCTTGTTC |
| JN982732 | GTTGATGATAGATGGTAGCGTTTTT | GGAGAGTCGAGTTATTTTGTGAGAG |
| JN982735 | CGTAAGATATCGGCACTCTTTACTC | AAGTAAGAACACCAATGATGAGTCC |
| JN982734 | CACCACGACCAGTATAATGTTGTTA | GAACAAGAAGAGTAAGGAGCACACT |
| JN982723 | AGTCTACTAGCAGCTGAGTCCATGT | GCCTCACCAACTAGCTAATCAGA |
